# Exploring the Potential of Heterosis to Improve Nitrogen Use Efficiency in Popcorn Plants

**DOI:** 10.3390/plants12112135

**Published:** 2023-05-28

**Authors:** Talles de Oliveira Santos, Antônio Teixeira do Amaral Junior, Rosimeire Barboza Bispo, Wallace de Paula Bernado, Bruna Rohem Simão, Valter Jário de Lima, Marta Simone Mendonça Freitas, Freddy Mora-Poblete, Roberto dos Santos Trindade, Samuel Henrique Kamphorst, Weverton Pereira Rodrigues, Eliemar Campostrini, Flávia Nicácio Viana, Cosme Damião Cruz

**Affiliations:** 1 Center for Plant Science Innovation, Department of Biochemistry, University of Nebraska—Lincoln, Lincoln, NE 68588-0664, USA; 2 Laboratory of Genetics and Plant Breeding, Center for Agricultural Sciences and Technologies (CCTA), Universidade Estadual do Norte Fluminense Darcy Ribeiro, Campos dos Goytacazes 28013-602, RJ, Brazil; rosimeirebarboza1@hotmail.com (R.B.B.); wallace-bernardo@hotmail.com (W.d.P.B.); rohembruna@gmail.com (B.R.S.); valter_jario@hotmail.com (V.J.d.L.); samuelkampho@hotmail.com (S.H.K.); campostrini@uenf.br (E.C.); flaalegre@hotmail.com (F.N.V.); cdcruz@ufv.br (C.D.C.); 3 Proteomics and Metabolomics Facilities, Nebraska Center for Biotechnology, University of Nebraska—Lincoln, Lincoln, NE 68588-0664, USA; 4 Plant Science Laboratory, Center for Agricultural Science and Technologies, Universidade Estadual do Norte Fluminense Darcy Ribeiro, Campos dos Goytacazes 28013-602, RJ, Brazil; msimone@uenf.br; 5 Institute of Biological Sciences, University of Talca, 1 Poniente 1141, Talca 3460000, Chile; fmora@utalca.cl; 6 National Research Center for Maize and Sorghum, Brazilian Agricultural Research Corporation, MG-424 Highway, Km 45, Sete Lagoas 35701-970, MG, Brazil; roberto.trindade@embrapa.br; 7 Centro de Ciências Agrárias, Naturais e Letras, Universidade Estadual da Região Tocantina do Maranhão (UEMASUL), Estreito 65975-000, MA, Brazil; wevertonuenf@hotmail.com

**Keywords:** gas exchange, genetic effects, Griffing diallel analysis, leaf pigments, nitrogen use efficiency, nutritional stress, *Zea mays everta*, sustainable agriculture

## Abstract

Nitrogen is crucial for plant growth and development, and improving nitrogen use efficiency (NUE) is a viable strategy for reducing dependence on nitrogen inputs and promoting sustainability. While the benefits of heterosis in corn are well known, the physiological mechanisms underlying this phenomenon in popcorn are less understood. We aimed to investigate the effects of heterosis on growth and physiological traits in four popcorn lines and their hybrids under two contrasting nitrogen conditions. We evaluated morpho-agronomic and physiological traits such as leaf pigments, the maximum photochemical efficiency of PSII, and leaf gas exchange. Components associated with NUE were also evaluated. N deprivation caused reductions of up to 65% in terms of plant architecture, 37% in terms of leaf pigments, and 42% in terms of photosynthesis-related traits. Heterosis had significant effects on growth traits, NUE, and foliar pigments, particularly under low soil nitrogen conditions. N-utilization efficiency was found to be the mechanism favoring superior hybrid performance for NUE. Non-additive genetic effects were predominant in controlling the studied traits, indicating that exploring heterosis is the most effective strategy for obtaining superior hybrids to promote NUE. The findings are relevant and beneficial for agro farmers seeking sustainable agricultural practices and improved crop productivity through the optimization of nitrogen utilization.

## 1. Introduction

In recent decades, world corn production has exponentially grown, and around 50% of this growth can be attributed to plant breeding and 50% to management practices, including nitrogen fertilization [1]. Regarding plant breeding, the exploitation of the heterosis effect has played a vital role in the extraordinary increase in corn yield around the world, especially in the United States of America, where grain yield has increased by 120% since the advent of the first hybrid in 1930 [2], becoming the largest producer of grain today. According to FAO [3], Brazil is the third largest corn producer in the world and, in 2019, became the largest exporter. In the 2019/2020 harvest, Brazilian production was around 103 million tons, of which approximately 23 million tons were exported [4].

In the last 20 years, despite the fact that the Brazilian corn yield has grown by around 120%, this value remains low (5.72 t ha^−1^) compared to the United States of America (11.07 t ha^−1^) and Argentina (8.36 t ha^−1^), countries where corn is grown in temperate and/or subtropical conditions and with a large amount of N application. In Brazil, even corn is grown in temperate and/or tropical conditions; around 75% of production comes from regions with a tropical climate, where the grain yield potential of cultivated areas is significantly reduced due to the occurrence of abiotic stresses [5,6,7]. Among the abiotic stresses, the low availability of nitrogen in the soil [8,9] is one of the main factors contributing to the grain yield reduction of several crops, including popcorn (*Zea mays* var. *everta*).

As an essential component of critical macromolecules, nitrogen is vital for plants [10], and in corn, it is an essential nutrient. N represents up to 5% of total dry matter [11] and is a constituent of leaf pigments, such as chlorophyll, amino acids, nucleic acids, proteins, and plant hormones [12,13]. The low availability of nitrogen in the soil directly impacts corn yield, with adverse effects on the weight and length of ears [14], 100-grain weight, prolificacy [15,16], as well as plant height [17]. These compromises in the agronomic components of production result from the effects of N limitation on photosynthetic capacity [18]. This is because the N supply has an important impact on carbon assimilation, considering that in a scenario of abiotic stresses, such as N deprivation, first response plants tend to promote the stomata closure, inhibiting the assimilation of CO_2_. 

Given its great importance in several physiological processes, nitrogen is the nutrient required in greater quantity to produce corn and is considered the second limiting factor for the increase in crop yield [9]. However, despite the high demand for N in corn production, only half of the applied N is used [19]. The remaining N is responsible for increasingly severe environmental pollution [20]. Therefore, given the scenario of increased adverse effects on agricultural sustainability, there is a need to develop a more sustainable agriculture model with genotypes efficient in the use of nitrogen [17] since the excessive use of this nutrient causes damage not only to the economy, but also to the environment. It must be considered that by 2050, agriculture will need to feed a growing world population, reaching the 10 billion mark [21].

For popcorn plants, which are highly appreciated in Brazil—and which moved to around US$10 billion worldwide in 2020 [22]—there have been few studies aimed at obtaining efficient genotypes in the use of N (NUE), as well as the exploration of heterosis for the release of more efficient hybrids in the use of the nutrient. This is because NUE is a complex trait controlled by several genes and highly influences the environment [23]. Therefore, understanding complex traits such as NUE requires a better understanding of the morphophysiological mechanisms underlying its expression, which is an arduous task from the point of view of plant breeding, mainly because it has already been reported that the components of NUE (N uptake efficiency—NUpE, and N utilization efficiency—NUtE) are independently inherited [24].

Aimed at understanding the mechanism of genetic control of NUE in popcorn, Santos et al. [25] evaluated the efficiency of nitrogen use in lines evaluated in two environments under adequate and infra-optimal conditions of nutrient availability in the soil. Subsequently, Santos et al. [26] evaluated the genetic effects involved in nitrogen use efficiency through the characterization of ten lines and ninety popcorn hybrids obtained in a complete diallel scheme for the two variables of interest for the crop—grain yield (GY) and popping expansion (PE). The authors’ conclusion was that both additive and non-additive effects contribute to the expression of NUE, along with the influence of the female parent, which is evident from the reciprocal effect’s significance. More recently, to understand the physiological mechanisms and proteomic profile of popcorn genotypes grown under different N availability conditions, Khan et al. [27] and Khan et al. [28] evaluated two contrasting lines for NUE together with their hybrid. According to the authors, the interaction between proteins related to the synthesis of L-ascorbate peroxidase and ferredoxin-nitrite reductase showed great importance in the expression of NUE for the species *Zea mays everta*.

However, studies on the morpho-physiological mechanisms associated with heterosis still represent a knowledge gap. Having access to additional knowledge regarding the genetic regulation of traits related to NUE expression, such as photosynthetic mechanisms, leaf pigments, and photochemical efficiency, would significantly assist in guiding breeding programs. This information would facilitate the reliable selection of parents and the production of superior hybrids, thereby playing a crucial role in the advancement of breeding programs. In this sense, evaluating lines and their hybrid combinations can provide relevant information for the popcorn breeding program [29]. Through these targeted crosses, estimates of general and specific combining abilities are obtained, which are associated with the additive and non-additive genetic effects involved in the control of traits [30,31]. In addition, through complete diallel, information can be obtained regarding reciprocal effects, which may be associated with the expression of extrachromosomal genes [26].

Although previous studies have shown that much of the nitrogen supply destined for the grain in maize is absorbed in the reproductive growth stage [32,33], the various negative effects of climate changes that intensified abiotic stresses caused a disruption in N assimilation and remobilization patterns [34]. In this sense, the assimilation of N in the vegetative stage is crucial to compensate for a possible deprivation of N in the grain-filling stage, mainly because between 45 and 65% of the N destined for the grains is provided by N remobilization with the advance of leaf senescence. Therefore, considering a scenario of water scarcity—common in countries with tropical and subtropical climates such as Brazil—genotypes that are more efficient in the use of N may present lower losses in productivity caused by N deprivation. From this perspective, Nasielski et al. [35] showed that a luxury N accumulation in the pre-anthesis period may be beneficial for plants since it is able to mitigate low N stress and act as an N reserve that buffers grain yield and maintains plant function.

Therefore, given the above, it was considered suitable to develop this study in which the goal is to evaluate the differences in growth, efficiencies of use, uptake, and utilization of nitrogen and the impacts of N starvation in the traits associated with photosynthetic efficiency. In addition, under contrasting conditions of nitrogen availability in the soil, the study aimed to understand the mechanisms involved in the expression of heterosis in popcorn genotypes and the genetic control of these traits under different N availability in plants in the vegetative stage. 

## 2. Results

### 2.1. Traits of Plant Architecture and Nitrogen Use Efficiency

Plant architecture traits, nitrogen content, and N use efficiency statistically differed between lines and hybrids, except for stem diameter and root N content in the high N condition. The Principal Component Analysis (PCA) showed that PC1 and PC2 accounted for more than 89% of the variance observed on the inbred lines in both N conditions (Supplementary Table S1). It could be observed that the NUE-related traits had the highest contributions for the two first PC’s in both N conditions (Supplementary Figure S1). For all growth traits, soil N deficit affected all evaluated traits, with significant interactions between genotypes and environments (G × N) being found (Table 1). Out of the 18 evaluated traits, the major contribution to the significant differences was given by the source of variation N (14 traits) (Supplementary Table S2).

Plant height, stem diameter, and leaf area were reduced by 8.6%, 33.4%, and 25.9%, respectively, by the reduction in soil N application. Regarding the dry matter, reductions of 60.0% and 53.6% were observed for the dry matter traits of the leaf and stem, respectively, and the reductions were 57.5% and 32.5% for the dry matter of the shoot and root, respectively (Table 1).

N deprivation had the biggest impact on the popcorn lines for plant height, stem diameter, and leaf area, which showed reductions of 12.3%, 38.4%, and 31.3%, respectively. In contrast, these reductions were 7.6%, 31.9%, and 24.5% for the hybrids, respectively. The same could be observed for the dry matter of leaf, stem, shoot, and root, which in the lines were reduced in the order of 65.6%, 62.4%, 64.4%, and 47.6%, while in the hybrids, the reductions were 58.6%, 51.4%, 55.8%, and 27.2%, respectively.

In this sense, the means of these traits (plant height, stem diameter, leaf area, dry matter of leaf, stem, shoot, and root) were higher in the hybrids, except for stem diameter at high N, whose contrast between lines and hybrids (C2) was not significant. The heterosis estimates for these traits were more marked in the limiting N condition. Thus, plant height, stem diameter, leaf area, and stem dry matter under N deficit presented estimates of 18.8%, 12.5%, 1.2%, 13.2%, and 39.2%, respectively. The shoot and dry root matter showed 23.2% and 49.4%, respectively (Table 1).

Regarding the N content in the plant, the joint analysis revealed a significant effect of the limitation of this nutrient in the genotypes studied, which caused decreases of 35.5%, 44.0%, 26.1%, 39.4%, and 36.8% in the N content in the leaf (LNC), stem (SNC), roots (RNC), shoot (STNC), and plant (PNC), respectively. Interestingly, for all these traits, the percentage decreases caused by the reduction of N in the soil were higher in the hybrids than in the lines. In the lines, the adverse effects of N reduction caused a decrease of 32.6%, 34.5%, 22.5%, 33.5%, and 31.1%, respectively, in LNC, SNC, RNC, STNC, and PNC. In the hybrids, the reductions in these traits were in the order of 36.3%, 46.4%, 27.6%, 41.0%, and 38.5%. Except for RNC, the nutrient reduction resulted in higher heterosis estimates in the high nitrogen supply environment for all traits, namely: 17.1% (LNC), 15.0% (SNC), 16.1% (STNC), and 12.3% (PNC) (Table 1). 

Regarding the estimates of N use efficiency, the interaction of genotypes with the environment was also significant (Table 1). However, the reduction in the N applied to the soil caused an increase in NUE, in the nitrogen uptake efficiency of the root with the N content (NUpE_cR), and in the nitrogen uptake efficiency without the N content (NUpE_sR). For these three traits, the increases caused in the means of lines and hybrids were 431.2%, 641.3%, and 614.9%, respectively. Considering that there was a significant difference (*p* ≤ 0.01) for contrast 2—lines and hybrids—(Table 1), the increase in these traits was more pronounced in the hybrids only for NUE (449.1% compared to a 361.2% increase in lines). Furthermore, for NUpE_cR and NUpE_sR, the most significant increases occurred in the lines, in the order of 699.2% and 675.2%, compared to 624.5% and 598.7%, respectively, in the hybrids.

Conversely, for the nitrogen utilization efficiency with N content in the root (NUtE_cR), as well as for the nitrogen utilization efficiency without N content in the root (NUtE_sR) and the nitrogen translocation efficiency (NTrE), reductions were observed caused by the reduced availability of the nutrient in the soil. For NUtE_cR and NUtE_sR, the reductions were more accentuated than those observed in NTrE (4.2%), with magnitudes of 29.8% and 32.7%, respectively, in the mean of the lines and hybrids. A significant difference (*p* ≤ 0.01) was detected between the lines and hybrids (C2). It appears that the reduction in these traits was more accentuated in the lines so that NUtE_cR and NUtE_sR were reduced by 47.0% and 48.7%, respectively, while in the hybrids this reduction was 24.4% and 27.8%, respectively. NTrE was much less affected in the lines and the hybrids, with respective values of 3.5% and 4.3%, but with a greater reduction in the hybrids (Table 1).

Given the differences observed between the performance of lines and hybrids for NUE in the N deficit conditions, the heterosis estimate was 23.2%, while in high N, the value was 14.0%. As for NUpE_cR and NUpE_sR, in low N, considering the inferior performance of the hybrids compared to the lines, the heterosis estimates were −9.6% and −6.7%, respectively, while in high N, the values were 12.3% and 16.1%, respectively, which reflects, in this case, the superior performance of the hybrids for these traits in this condition.

For NUtE_cR, NUtE_sR, and NTrE, the heterosis estimates were more modest, with the limiting soil N supply environment being responsible for the highest estimates, in percentages of 33.4%, 36.3%, and 3.1%, respectively. These values were 1.1%, 4.6%, and 3.4% in the high N environment.

For the two N supply conditions, the importance (expressed in %) of the quadratic components pertaining to the general combining ability (ϕ_g_), specific combining abilities (ϕ_s_), and the reciprocal effects (ϕ_rc_) of the traits associated with plant architecture, the status of N in the plant, and the efficiencies in the use, uptake, and utilization of N. It was observed that the general (related to ϕ_g_) and specific combining ability (related to ϕ_s_) differed in the two N supply conditions for all traits (Supplementary Table S3).

**Figure 1:**
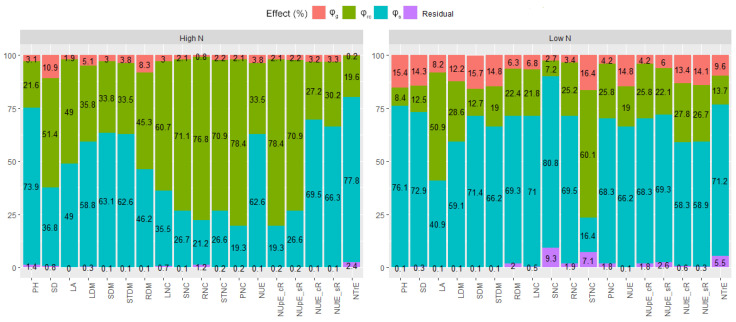
Importance in % of quadratic components related to general (ϕ_g_) and specific (ϕ_s_) combining abilities and reciprocal effects (ϕ_rc_) for plant architecture traits (PH—plant height; SD—stem diameter; LA—leaf area; LDM—leaf dry matter; SDM—stem dry matter; STDM—shoot dry matter; RDM—root dry matter; LNC—leaf N content; SNC—stem N content; RNC—root N content; STNC—shoot N content; PNC—plant N content; NUE—nitrogen use efficiency with (cR) and without (sR) root N content; NUpE—nitrogen uptake efficiency with (cR) and without (sR) root N content; NUtE—nitrogen utilization efficiency with (cR) and without (sR) root N content; and NUtrE—nitrogen translocation efficiency.

Although the mean squares related to the quadratic components ϕ_g_ were significant at high N, the essential components to explain the observed genetic variability for growth traits, N status, and nutrient use efficiency were those related to the specific combining ability (ϕ_s_) and reciprocal effects (ϕ_rc_). Therefore, for the traits PH, LDM, SDM, STDM, RDM, NUE, NUtE_cR, NUtE_sR, and NTrE, the prevalence of non-additive genetic effects was evident. In the case of the LA, the components ϕ_s_ and ϕ_rc_ had values very close to the relative contribution, with estimates of 49.01% and 49.02%, for ϕ_s_ and ϕ_rc_, respectively. These reciprocal effects were more important for SD, LNC, SNC, RNC, STNC, PNC, NUpE_cR, and NUpE_sR (Figure 1, Supplementary Table S2).

**Figure 2:**
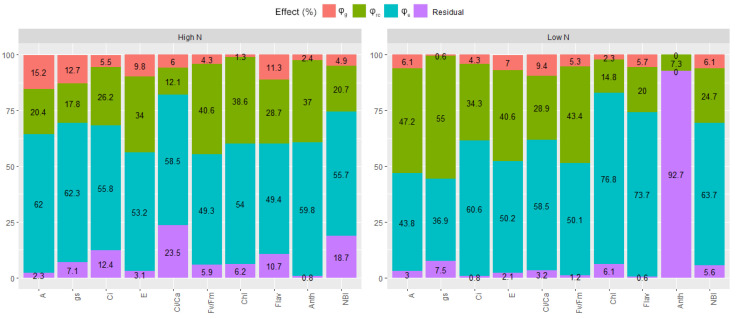
Importance (expressed in %) of the quadratic components related to general (ϕ_g_) and specific combining abilities (ϕ_s_) and of reciprocal effects (ϕ_rc_) for traits related to photosynthesis, leaf pigments and photochemical efficiency of chlorophyll (A—net CO_2_ assimilation rate; gs—stomatal conductance; Ci—intercellular concentration of CO_2_; E—transpiration rate; Ci/Ca—ratio between the intercellular and external concentration of CO_2_; Fv/Fm—maximum quantum efficiency of photosystem II; Chl—relative chlorophyll content; Flav—relative content of flavonoids; Anth—relative content of anthocyanins; and NBI—nitrogen balance index).

In soil N-limiting conditions, for most traits, there was a predominance of the contribution of the quadratic component associated with non-additive effects (ϕ_s_) in the expression of genetic variability (Figure 2 and Supplementary Table S3). In this sense, it could be observed that for 16 of the 18 traits related to growth, N status in the plant and nutrient use efficiency—PH, SD, LDM, SDM, STDM, RDM, LNC, SNC, PNC, NUE, NUPE_cR, NUpE_sR, NUtE_cR, NUtE_sR, and NTrE—the quadratic component ϕ_s_ presented greater contributions. For LA and STNC, it was possible to observe a greater contribution of the quadratic component associated with the reciprocal effect (ϕ_rc_). It is important to highlight that for the two instances of N availability in the soil, the residual effects were not very expressive and, therefore, of minor importance for the observed results, guaranteeing an unequivocal interpretation of the observed effects (Figure 1 and Supplementary Table S3).

### 2.2. Gas Exchange, Photochemical Efficiency of Chlorophyll, and Leaf Pigments Measurements 

The joint analysis of variance revealed a significant effect of nitrogen limitation on the genotypes—means of lines and hybrids — with a decrease in the net CO_2_ assimilation rate (A) of 28.4%, in addition to reductions in stomatal conductance (gs) of 36.1%, in the intercellular concentration of CO_2_ (Ci), and in the ratio between the intercellular and external concentration of CO_2_ (Ci/Ca) of 12.8% and 12.5%, respectively, as well as the transpiration rate (E) of 18.7% (Table 2). In terms of the contribution to significative differences observed, for the gas exchange and related traits and chlorophyll fluorescence, the effect of genotype and N condition were equally relevant, being the G × N interaction, relevant just for one trait (Supplementary Table S1). In addition to the effects between the genotypes, it could be seen that the lines presented smaller percentage reductions concerning the hybrids and that there was an increase, even if not very expressive—of a magnitude of 0.3%—for the transpiration rate. In this sense, for the lines, the reductions caused in A, gs, Ci, and in the Ci/Ca ratio were 19.0%, 15.1%, 6.0%, and 0.6%, respectively. For the hybrids, the reductions for the same traits were 31.4% (A), 41.5% (gs), 14.9% (Ci), and 14.0% (Ci/Ca). However, for the maximum photochemical efficiency of photosystem II (F_v_/F_m_), with the imposition of stress, there was an increase of 5.7% in the average of the lines and hybrids; however, this increase was mainly due to the increase of 7.9 % in the average of the hybrids, while in the lines there was a negative impact of 0.6% (Table 2). 

In the two conditions of N availability in the soil and considering that there is a significant difference (*p* ≤ 0.01) in the contrast between lines and hybrids for A, *gs*, E, and Ci/Ca, the heterosis estimates in the high nitrogen condition environment were higher for A, *gs*, E, and Ci/Ca, with estimates of 17.5%, 33.9%, 28.4%, and 21.9%, respectively. For F_v_/F_m_, under the condition of high N in the soil, there was a significant difference between lines and hybrids, which resulted in a reduced heterosis value (−1.1%). For traits A, g_s_, E, Ci/Ca, and F_v_/F_m_, in N limiting condition, heterosis estimates were 1.0% (A), −4.3% (*gs*), −9.7% (E), 20.3% (Ci/Ca), and 10.1% (F_v_/F_m_). For Ci, a higher estimate for heterosis was obtained in the low N condition, of 3.5%, while in the high N condition, the estimate was 2.6% (Table 2).

Regarding leaf pigments, except for the relative content of anthocyanin (Anth) in high N, a significant difference was found between the genotypes studied, in addition to a significant interaction between genotype and N condition (G × N), with N being the source of variation, the one that most influenced the significant differences observed. In this sense, it could be observed that nitrogen limitation had an impact on the relative content of chlorophyll (Chl), flavonoids (Flav), and the nitrogen balance index (NBI, Chl/Flav ratio). In the relative content of chlorophyll, under N-limited conditions, there was a reduction of 24.1%. In comparison, it caused an increase of 9.5% and 37.1% in the relative contents of the accessory pigments, anthocyanins, and flavonoids, respectively. Regarding the nitrogen balance index, there was a reduction of 31.1% based on the average performance of the lines and hybrids (Table 2).

Taking into consideration the significance of the contrast between lines and hybrids, it is notable that the reductions observed in the relative chlorophyll content and nitrogen balance index were more prominent in the hybrids. Specifically, the decrease in relative chlorophyll content caused by soil nitrogen limitation was 25.6% in hybrids, while the reduction in the nitrogen balance index was 33.4%. In contrast, the reductions in these traits for the lines were 18.8% (chlorophyll) and 23.6% (nitrogen balance index). On the other hand, the hybrids exhibited more substantial increases in accessory pigments. The estimated percentage increase was 10.6% for the relative content of flavonoids and 44.0% for the relative content of anthocyanins (Table 2).

In heterosis estimates, nitrogen limitation resulted in more expressive values. At low N, the values for Chl, Flav, and NBI were −10.5%, 10.2%, and −18.4%, respectively. Therefore, no significant difference was observed in contrast between lines and hybrids in this condition. In high nitrogen conditions, a significant difference (*p* ≤ 0.01) was observed between lines and hybrids only for anthocyanin content, the heterosis estimate of which was −2.7% (Table 2). 

Despite the significance observed in the mean squares of general combining ability, specific combining ability, and reciprocal effects for gas exchange measures, photochemical efficiency of chlorophyll, and leaf pigments in both nitrogen supply conditions, the ϕs component associated with non-additive genetic effects was predominant (Figure 2, Supplementary Table S3). Notably, a substantial contribution of residual effect was observed for the relative content of anthocyanin in low N conditions, indicating great environmental influence. Consequently, negative estimates for the quadratic components ϕ_g_ and ϕ_s_ were obtained. In this context, these negative values are interpreted as estimates with a true magnitude equal to zero. Therefore, the quadratic component was not considered further, as it accounted for 0% of the variation and did not explain the genetic variability of the trait

## 3. Discussion

### 3.1. The Effect of Nitrogen Deprivation on Photosynthesis, Maximum Efficiency of PSII, Leaf Pigments, and Its Impact on the Growth of Popcorn Genotypes

Nitrogen plays a vital role in plants [18] and, in leaves, nitrogen forms include soluble components such as nitrate, amino acids, and proteins, as well as insoluble constituents in cell walls and membranes, among other structures [36]. The nitrogen utilized by the photosynthetic apparatus can be categorized into two main types: (i) nitrogen associated with enzymes involved in CO_2_ assimilation, and (ii) nitrogen present in thylakoids and associated with photochemical efficiency [37]. In terms of the nitrogen’s association with enzymes, it is found in the structure of key enzymes such as ribulose-1,5-bisphosphate carboxylase (Rubisco), phosphoenolpyruvate carboxylase (PEPC), and pyruvate orthophosphate dikinase (PPDK). These enzymes play a direct role in the reduction reactions of carbon and are the most abundant enzymes involved in the assimilation of CO_2_ [38]).

In relation to nitrogen associated with thylakoids, this nutrient can be divided between two types of proteins. The first type includes proteins involved in bioenergetics, such as Cyt b6f and CF1/CF0, which play roles in electron transport and phosphorylation [39,40]. The second type of protein is associated with the light-harvesting complexes II (LHCII) and I (LHCI) [41]. 

In plants with C4 metabolisms, such as popcorn, approximately 45% of the nitrogen is allocated to soluble proteins, with 20% of this portion being attributed to Rubisco. Another 28% of nitrogen is allocated to thylakoids. Within the thylakoids, approximately 75% of the nitrogen is associated with light-harvesting proteins, while the remaining portion is dedicated to bioenergetics [38]. Consequently, a low supply of nitrogen has a negative impact on the photosynthetic process, ultimately affecting plant development. 

Based on the results of the present work, during vegetative growth, N limitation in popcorn plants caused significant reductions in plant height, stem diameter, and leaf area (Table 1). These reductions may be mainly associated with the decrease observed in traits related to photosynthesis, such as net photosynthetic rate (A), stomatal conductance (gs), intercellular CO_2_ concentration (Ci), transpiration (E), and the ratio between the intercellular and external concentration of CO_2_ (Ci/Ca), which were reduced by the magnitudes of 28.4%, 36.1%, 12.8%, 18.7%, and 12.5%, respectively (Table 2).

The reduction in the traits associated with photosynthesis caused by the N limitation in the soil was more expressive in the hybrids than in the lines. However, the hybrids presented higher values for plant height, stem diameter, and, mainly, leaf area in this N limitation condition (Table 1). The larger leaf area is essential to increase the photosynthetically active radiation (PAR) interception area and, therefore, to increase CO_2_ assimilation—under the condition of adequate stomatal conductance values—and transpiration in the plant [38]. Although under soil N limiting conditions, the hybrids reduced the estimates of A, gs, E, and chlorophyll contents by −1.4, −4.3, −9.7, and −10.5%, respectively, these decreases were not enough to cause reductions in the growth variables (Table 2). On the contrary, in the N-limiting condition, the hybrids increased the estimates related to the growth traits (Table 1). In this way, the hybrids produced a greater amount of plant dry matter with a smaller amount of assimilated CO_2_ (lines ≅ 7.9 g of shoot dry matter per µmol of CO_2_ assimilated; hybrids ≅ 9 g of shoot dry matter per µmol^−1^ CO_2_ assimilated), if we could consider a hypothetic scenario where the responses found on the V6 leaf could be extrapolated to the photosynthesis of the whole plant.

In the condition of reduced CO_2_ assimilation, the excitation energy surplus due to the decrease in ATP and NADPH consumption promotes an increase in the susceptibility of PSII to the action of photons on this photosystem (photoinhibition) [42,43,44] and PSII damage can compromise the biomass production of plants. However, this did not happen with the hybrids since, in the condition of N limitation in the soil, the F_v_/F_m_ ratio values expressed an increase of 10% concerning the lines. This tolerance of the hybrids may be associated with the reduction in the concentration of chlorophylls in the leaves. The reduction in the relative chlorophyll content in the hybrids was higher than in the lines, with an estimate of 25.6%, when compared to the value of 18.8% in the lines. According to Khamis et al. [45] and Lu et al. [43], reducing the relative chlorophyll content may be a strategy to protect the PSII function since it can avoid the excessive production of excitation energy, which could cause damage to the PSII.

The F_v_/F_m_ ratio, which makes it possible to verify whether there was damage to the photosynthetic apparatus, is a variable that represents the maximum photochemical efficiency of PSII in a condition in which all reaction centers are open and receive a pulse of light saturating. Under stress conditions, such as the reduction of N availability in the plant, there may be a decline in the values of this trait, which indicates possible damage to the photochemical machinery [46,47]. Therefore, genotypes that present tolerance mechanisms to protect the PSII may show insignificant reductions or even higher values for this measure, even in stressful situations [48,49,50]. Therefore, as observed in the hybrids under N-limited conditions, it can be suggested that the regulatory mechanisms associated with the reduction in the total chlorophyll content favored the elevation of the F_v_/F_m_ ratio in the hybrids. Based on the results, it can be inferred that the hybrids were more efficient in the assimilation of CO_2_ per unit of chlorophyll molecules (Table 2) than the lines, which was reflected in the greater capacity to allocate the photoassimilates produced in the production of matter drought, generating positive impacts on the total dry matter of the plant (Table 1).

Among the genotypes evaluated, the hybrids showed essential increases in the relative content of flavonoids (Flav), which are phenolic compounds associated with the adaptive responses of plants to various abiotic stresses, such as drought [51], the reduced availability of nutrients in the soil, and the excess of solar radiation [52,53]. These compounds mainly act as accessory pigments of chlorophyll molecules, protecting against reactive oxygen species (ROS), which can have high rates in plants under suboptimal N conditions. ROS can degrade plant cells through the oxidation of membranes [54] and the degradation of molecules, such as DNA [55]. The production of these ROS is mainly associated with the reduction of stomatal conductance, which is regulated by the action of ABA through stress signaling from the roots [56]. Therefore, in the hybrids, when compared to the lines, the notable increase in secondary metabolites such as flavonoids (Flav) and anthocyanins (Anth) could potentially provide an adaptive advantage over the parent plants. This increase in secondary metabolites might have contributed to improved physiological and agronomic performance.

### 3.2. The Mechanisms Underlying the Efficient Use of N in Popcorn Genotypes

Efficiency in the use of N and associated components (i.e., efficiency in uptake and utilization) was proposed by Moll et al. [57]. The authors defined N use efficiency (NUE) as the ratio between the grain weight and the available N in the soil or the product of the N uptake efficiency (NUpE: the ratio between the total N in the plant and the available N in the soil) and the N utilization efficiency (NUtE: the ratio between the weight of grains and the total N in the plant). Subsequently, Good et al. [58] proposed that NUE is the ratio between dry shoot matter and applied N. NUpE is the ratio between the N content in the plant and the amount of N applied to the rhizosphere, and NUtE is the ratio between the shoot dry matter and the N content in the plant. However, when assessing the efficiency in the uptake and utilization of N, it remains uncertain whether the N content of roots should be included since some authors only use the N content in the shoot [59,60] and others consider the N content of the plant, including the N content of the roots [37,57,61,62].

Given the significant differences between the genotypes for NUE and the components associated with this variable (with and without the assessment of N content in the root), under high and low N conditions, it can be noted that when considering the N content in the roots, it is possible to obtain more reliable estimates of N uptake and utilization since, up to the V6 stage, the N content in the root has a crucial role for the growth of the plants.

Regarding the effect of N reduction, a significant increase in NUE was observed in popcorn genotypes under N-limiting conditions. This increase could also be observed for NUpE_cR and NUpE_sR. For NUtE_cR, NUtE_sR, and NTrE, reductions were observed when the soil had limited nitrogen. In this condition, corn shows an increase in N use efficiency (NUE), either through increased uptake (NUpE) or N utilization (NUtE) [63,64]. As N is quite mobile and found in deeper soil profiles [65], the increase in nitrogen uptake may be associated with an increase in area and a deepening of the root system [66]. NUtE can be defined as the amount of dry matter produced per unit of N in the plant; the increase in the value of this variable is an indicator of how efficiently the plant can use the available N in the photosynthetic machinery [17]. 

With the N limitation in the soil, the lines had more significant reductions in NUE, while the hybrids stood out with higher values for this trait. However, in this study, the most important mechanism to increase the NUE of the hybrids was the use and transport of the nutrient since the hybrids presented more expressive values compared to the lines. This becomes clear when the N contents in different plant tissues are compared in the limiting N condition. In this condition, the hybrids presented higher N content in the leaves, while the lines concentrated most N in the stem and root (Table 1). Therefore, even with a higher net photosynthetic rate and higher relative chlorophyll content, the lines were not efficient in using N to increase their leaf area and dry matter; however, they were more efficient in the acquisition of the nutrient, considering that in the condition limiting the values for N content in the plant and NUpE were higher in the lines. 

### 3.3. What Is the Best Strategy for Conducting Popcorn Breeding to Increase the Nitrogen Use Efficiency?

In general, the N limitation in the soil caused greater discrimination between the genotypes studied and resulted in higher heterosis estimates in this environment. This is associated with the fact that in this condition, the genotypes have greater differentiation for the traits studied, which has already been reported by other studies with common corn [67,68] and popcorn [27]. Therefore, the selection of genotypes in this condition may be more effective in obtaining a high genetic variance. Furthermore, based on the estimates of the mean squares and the quadratic components associated with the general and specific abilities of combination and the reciprocal effect, it was evidenced that there is no difference between the environments regarding the quadratic component that contributed most to the genetic variance of the studied traits. The genetic mode of action is the same for both conditions—high and low N, which may allow the use of the same breeding strategies in both conditions. 

For the traits associated with growth—plant height, dry matter of leaf, stem, shoot, and root—in the two N conditions, the contribution of the quadratic component associated with non-additive genetic effects (ϕ_s_) predominated. This fact indicates that the exploitation of heterosis is recommended to achieve genetic gains [30,31]. Although ϕ_s_ was essential for most traits, some showed a greater predominance of the quadratic component associated with reciprocal effects, as is the case of stem diameter in high N and leaf area in both N conditions. In addition, the genetic effects associated with the reciprocal effect prevailed for the N contents in the leaf, stem, root, shoot, and mining plant. The same could not be observed in low N conditions, prevailing for these traits—except N content in the shoots—the genetic effects associated with ϕ_s_. However, even with the predominance of genetic effects associated with ϕs, the other components also showed significance, indicating that there is, even to a lesser degree, the influence of additive genetic effects (ϕ_g_) and the female parent (ϕ_rc_). This fact indicates that, based on the selection of genotypes for NUE, the female parent should be the one with the best values for the evaluated trait. This can be explained by the fact that the mechanisms underlying better performance for NUE, such as the photosynthetic response and the content of leaf pigments, are determined by the female parent through chloroplasts located in the cytoplasm of the female gamete [69].

For the variables evaluated for the general and specific combining ability, as well as for the reciprocal effect on the two levels of N used, the significance of the interactions allows us to confirm that the alleles that control the expression of traits under low nutrient supply are partially different from those that control the same traits under optimal nutrient supply [26]. This implies that the performance of genotypes under low N conditions availability involves genes expressed under optimal N availability and that other genes are expressed or silenced [70]. 

## 4. Materials and Methods

### 4.1. Plant Material and Growth Conditions

Four popcorn lines (S_7_)—P2 (derived from the compound CMS-42, adapted to temperate/tropical climates), P7 (derived from the hybrid IAC112, adapted to temperate/tropical climates), L75 and L80 (derived from the open pollination variety Viçosa, adapted to temperate/tropical climates)—and the hybrids, including reciprocal combinations, were evaluated under contrasting conditions of N availability. The lines were selected based on previous studies under contrasting N conditions in the soil and classified as efficient (P2 and P7) and inefficient (L75 and L80) in nitrogen use [25,26]. Following the order of female and male parents, the hybrids P2 × P7, P2 × L75, P2 × L80, P7 × P2, P7 × L75, P7 × L80, L75 × P2, L75 × P7, L75 × L80, L80 × P2, L80 × P7, and L80 × L75 were used.

The experiment took place in a protected cultivation environment within a greenhouse at the Experimental Support Unit of the Universidade Estadual do Norte Fluminense Darcy Ribeiro (21°9′ S; 41°10′ W, 14 m altitude) from 10 March to 20 April 2021. A lysimetric system, following the description by Elazab et al. [71], was utilized for the experiment. The system consisted of polyvinyl chloride (PVC) tubes with a diameter of 15 cm and a length of 150 cm, which were cut in half lengthwise. The two halves of the tubes were securely fixed together with adhesive tape. The lower parts of the tubes were sealed with pots of the same diameter, enabling proper drainage. The tubes, known as lysimeters, were filled with sand that had been washed using deionized water. Before commencing the experiment, samples of the substrate were collected and subjected to chemical analysis to evaluate the nutrient availability. The results of the chemical analysis can be found in Supplementary Table S4.

**Figure 3:**
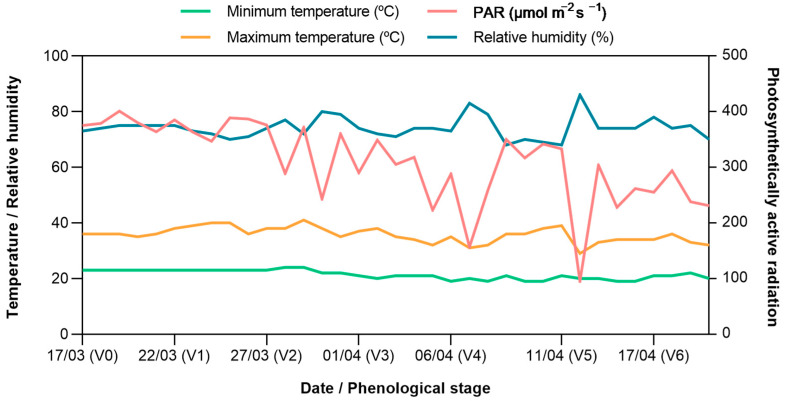
Means of minimum and maximum temperatures (°C), relative humidity (RH, %), and photosynthetically active radiation (PAR, µmol m^−2^ s^−1^) during the dates and phenological stages (V) of popcorn plant growth under two N availability conditions (March to April 2021).

The experiment was arranged in complete randomized blocks, under two nitrogen availability conditions, with three replications per genotype for each nutrient availability in a factorial arrangement. Three seeds per lysimeter were sown, and after thinning (10 days after germination), only one plant per tube was maintained for each genotype (one plant per biological replicate). The spacing between plants was 25 cm and between rows was 1 m (40,000 plants ha^−1^). The temperature, humidity, and photosynthetically active radiation data followed the seasonal pattern and were obtained using the WatchDog 2000 Series Experimental Station (Spectrum Technologies Inc., Aurora, IL, USA) (Figure 3).

The two nitrogen (N) availability conditions were based on 100% N (control condition, 224.09 mg L^−1^) and 10% N (treatment, 22.41 mg L^−1^), as established by Khan et al. [27], as being the ideal contrasting N levels to better discriminate popcorn genotypes for NUE and other morphophysiological traits. The solutions used were based on the modified Hoagland and Arnon [72]. The plants were irrigated daily with deionized water, and nutrients were supplied from the V2 stage (two fully expanded leaves), applying 200 mL of the nutrient solution with a pH between 5.5 and 5.8. Two contrasting dosages of N were used: 100% N requirement and 10% N requirement (22.41 mg L^−1^) (90% reduction in soil N availability).

### 4.2. Leaf Pigments

The measurements of chlorophyll, flavonoids, anthocyanins, and the nitrogen balance index were conducted on the middle third of the sixth fully expanded leaf (V6) one day prior to the conclusion of the experiment, which was 30 days after sowing. A portable meter called Dualex^®^ (manufactured by FORCE-A, Orsay, France) was used for these measurements. The analysis was performed at the specific location on the leaf where chlorophyll fluorescence emission and gas exchange were evaluated.

### 4.3. Chlorophyll Fluorescence Measurements

Chlorophyll fluorescence was evaluated one day before the end of the experiment (V6 stage), in the middle third of the last fully expanded leaf of each plant from 9:00 to 11:00 h, using the Pocket PEA fluorimeter (Hansatech, King’s Lynn, UK). The leaf was adapted in the dark for 20 min before using the leaf clip. Then, the leaf samples were exposed to a saturating light pulse (3500 μmol m^−2^ s^−1^) to evaluate the maximum quantum efficiency of PSII (F_v_/F_m_).

### 4.4. Measurements of Leaf Gas Exchange

Gas exchange [net photosynthetic rate (A), stomatal conductance (gs), and transpiration rate (E)] was evaluated one day before the end of the experiment, in the middle third of the sixth fully expanded leaf of each plant (V6), between 09:00 and 11:00 h, and an infrared gas analyzer, model LI-6400 (LI-COR, Lincoln, NE, USA), equipped with a 6 cm^2^ leaf chamber and an external light source (6400–40 LCF, LI-COR). During the evaluations, the PPFD was set at 1500 µmol m^−2^ s^−1^, the CO_2_ concentration was 400 µmol mol^−1^, the relative humidity was between 55% and 60%, and the temperature was situated at 25 °C.

### 4.5. Morphological Traits 

At the end of the experiment, the plant height (cm) was measured with a ruler from the tube surface to the last developed leaf (visible ligule). The stem diameter, quantified in mm, was measured at the height of the middle third of the plants. The leaf area (cm^2^) was obtained through the product between the width (cm) and length of the leaf (cm) with a tape measure. Then, the leaves were separated from the stems and placed in paper bags for drying in a circulation oven at 65 °C for 72 h to determine the dry matter of the leaf (LDM—g) and stem (SDM—g) on a scale of precision. The shoot dry matter (STDM—g) was obtained by adding the LDM and SDM. 

### 4.6. Root System Analysis 

At the end of the experiment, the tubes were opened to separate the substrate from the roots. Then, the samples were gently shaken and washed with running water, using a screen to remove the soil. Root samples were washed with deionized water, lightly dried with paper towels, and placed in a paper envelope. Then, they were taken for drying in an oven at 65 °C for 72 h to determine the dry matter (RDM—g) on a precision balance.

### 4.7. Concentration of N

The N analysis was determined using the Kjeldahl method [73], obtaining the N content in the leaves (LNC: LDM × N content in the leaf—mg), in the stem (SNC: SDM × N in the stem—mg), in the shoot (STNC: STDM × N content in the shoot—mg), in the root (RNC: RDM × N content in the root—mg), and in the plant (PNC: sum of STNC and RNC—mg).

### 4.8. Efficiency in the Use of Nitrogen and Components

With the information on N application in the soil and the dry matter produced, the N use efficiency (NUE: STDM/total applied N), the N uptake efficiency with the root N content (NUpE_cR: PNC/N total applied), the N uptake efficiency without the root N content (NUpE_sR: STDM/total applied N), the N utilization efficiency with the root N content (NUtE_cR: STDM/N content in the plant), the N utilization efficiency without the content of N of the root (NUtE_sR: STDM/N content in the shoot), and the N translocation efficiency (NTrE: STNC/N content in the plant).

### 4.9. Heterosis Estimation

For each trait, heterosis (H) was calculated by the difference between the average value obtained by the hybrid (F1) regarding the average values obtained by its parents (MP), in absolute and percentage values, respectively, according to the expressions: $MP=\frac{P1+P2}{2}$ and $H=\left(\frac{F1-MP}{MP}\right)\times 100$; where P1 and P2 refer to the averages of the parents and F1 refers to the average performance of the hybrid [30]. 

### 4.10. Statistical Analysis

For each trait studied, an individual analysis of variance was performed for each nitrogen availability condition according to the following statistical model: $Y_{ij}=\mu +G_{i}+B_{j}+\varepsilon_{ij,}$ where $Y_{ij}$ is the observed value of the i-th genotype in the j-th block, μ is the general constant, $G_{i}$ is the effect attributed to the i-th genotype, $B_{j}$ is the effect of block j, and $\varepsilon_{ij}$ is the experimental error associated with the observation $Y_{ij}.$

Subsequently, a joint analysis of variance was performed based on the following statistical model: $Y_{ijk}=\mu +B_{k}+G_{i}+A_{j}+{GA}_{ij}+\varepsilon_{ijk},$ where $Y_{ijk}$ is the observation of the i-th genotype in the j-th availability of N in the k-th block, μ is the general constant, $G_{i}$ is the fixed effect of the i-th genotype, $B_{k}$ is the random effect of the k-th block, $A_{j}$ is the fixed effect of the j-th condition of N, ${GA}_{ij}$ is the fixed effect of the interaction between the i-th genotype with the j-th condition of N, and $\varepsilon_{ijk}$ is the average experimental random error associated with the observation $Y_{ijk}$ with NID (0, $\sigma^{2}$). The differences between lines and hybrids were partitioned for each trait, considered as contrast I (C1—differences between lines), contrast 2 (C2—differences between lines and hybrids), and contrast 3 (C3—differences between hybrids). Statistical analyzes were performed using SAS 9.4 software (SAS Institute Inc., Cary, NC, USA). 

The combinatorial abilities were analyzed by the method I of diallel analysis proposed by Griffing (1956), in which the parents, the hybrids, and the reciprocals are evaluated, considering the effect of the fixed genotypes. The effects of the genotypes for the general combining ability (GCA) and the specific combining ability (SCA) were obtained considering the following model: $Y_{ij}=µ+\;g_{i}+g_{j}+\;s_{ij}+\;r_{ij}\varepsilon_{ij}$, where $Y_{ij}$ is the average value of the hybrid combination (i ≠ j) or the parent (i = j), µ is the overall average, $g_{i}$, $g_{j}$ are the effects of the general combining ability of the i-th or j-th parent (i, j = 1, 2, 3, and 4), $s_{ij}$ is the effect of the specific combining ability for crosses between parents of order i and j, $r_{ij}$ is the reciprocal effect that quantifies the differences resulting from parent i or j when used as a male or female parent in the cross ij, and $\varepsilon_{ij}$ is the average experimental error associated with the observation of order ij.

The quadratic components that express the genetic variability associated with GCA (ϕ_g_), SCA (ϕ_s_), and reciprocal effects (ϕ_rc_) were estimated by: $\phi g=\frac{QMG-QMR}{2p}$, ϕs=QMS−QMR, and $\phi rc=\frac{QMRC-QMR}{2}$, where QMG is the mean square of the general combining ability, QMS is the mean square of the specific combining ability, QMRC is the mean square of the reciprocal effect, QMR is the mean square of the residual, and p is the number of parents. To test the importance (R^2^) of the Sources of Variation genotypes (G), N condition (N), and G by N interaction (G × N) for each trait, we estimated the ratio between the sum of square (SQ) of a given trait and its total SQ (SQT). Therefore, we obtained the following parameters: R^2^_G_ (proportional contribution of G), R^2^_N_ (proportional contribution of N), and R^2^_G×N_ (proportional contribution of G × N).

The effects of the quadratic components were expressed as percentages concerning the sum of the total effects. Statistical-genetic analyzes were performed using the Genes software [74]. Finally, the PCA was performed on RStudio [75] using the package FactoMineR [76].

## 5. Conclusions

In popcorn, in the vegetative stage, the effects of heterosis related to plant biomass resulted in higher production of shoot dry matter. Regarding the parents, the better performance of the hybrids was even more evident in conditions of low N availability in the soil, in which it was established that the adaptation of *Zea mays everta* to environments with N deficiency requires the exploitation of hybrid vigor. 

In the hybrids, under limiting conditions of N in the soil, contrary to what was expected, there was a greater reduction in leaf gas exchange, which was not enough to reduce the growth of these genotypes, guaranteeing higher estimates for NUE, promoted by better use of the nitrogen available.

It is suggested that under limiting N condition, the adaptive mechanisms developed by the hybrids were the reduction of the total chlorophyll content and the increase in the levels of accessory pigments—anthocyanins and flavonoids—which could improve the protection of the photosynthetic apparatus and higher maximum photochemical efficiency of the PSII.

Future perspectives based on the results found points in two directions: (i) conducting comparative proteomics and mRNA-sequencing studies to comprehend the molecular mechanisms underlying the NUE response in the most contrasting inbred lines; and (ii) examining the evaluated genotypes (lines and hybrids) under field conditions throughout the crop development cycle to gain insights into the impacts of N starvation on traits such as grain yield and popping expansion.

## Figures and Tables

**Table 1:** Summary of joint and individual ANOVA, means and standard deviations of morpho-physiological traits, nitrogen (N) content, and N use efficiency of lines and diallel hybrids of popcorn cultivated under contrasting conditions of N availability.

Trait	Joint Analysis			High N Condition						Low N Condition					
	G	N	G × N	Lines	C1	Hybrids	C2	C3	H%	Lines	C1	Hybrids	C2	C3	H%
PH	**	**	**	28.28 ± 3.30	**	31.48 ± 4.60	**	**	14.5	24.80 ± 3.01	**	29.09 ± 4.62	**	**	18.8
SD	**	**	**	10.42 ± 1.57	**	10.52 ± 1.64	^ns^	**	6.9	6.42 ± 1.06	**	7.17 ± 0.91	**	**	12.5
LA	**	**	**	233.37 ± 22.23	**	226.62 ± 49.22	**	**	−2.2	160.29 ± 21.92	**	172.02 ± 25.03	**	**	1.2
LDM	**	**	**	4.27 ± 1.00	**	3.77 ± 1.23	**	**	−8.9	1.47 ± 0.53	**	1.57 ± 0.53	**	**	13.2
SDM	**	**	**	2.55 ± 0.57	**	2.49 ± 1.07	**	**	−0.9	0.96 ± 0.34	**	1.21 ± 0.44	**	**	39.2
STDM	**	**	**	6.82 ± 1.57	**	6.29± 2.21	**	**	−5.2	2.43 ± 0.85	**	2.78 ± 0.92	**	**	23.2
RDM	**	**	**	1.03 ± 0.42	**	1.06 ± 0.35	**	**	23.7	0.54 ± 0.05	**	0.77 ± 0.25	**	**	49.4
LNC	**	**	**	25.55 ± 1.39	**	28.51 ± 3.86	**	**	17.1	17.23 ± 3.72	**	18.15 ± 2.64	**	**	1.1
SNC	**	**	**	22.75 ± 1.51	**	24.62 ± 5.03	**	**	15.0	14.90 ± 1.62	**	13.19 ± 1.42	**	**	−11.3
RNC	**	**	**	13.23 ± 0.26	^ns^	12.41 ± 2.45	**	**	−1.4	10.26 ± 0.68	**	8.99 ± 0.90	**	**	−18.1
STNC	**	**	**	48.30 ± 2.30	**	53.13 ± 8.55	**	**	16.1	32.13 ± 2.42	**	31.34 ± 3.40	**	**	−6.7
PNC	**	**	**	61.53 ± 2.39	**	65.54 ± 10.79	**	**	12.3	42.39 ± 2.05	**	40.33 ± 4.01	**	**	−9.6
NUE	**	**	**	124.15 ± 28.51	**	114.50 ± 40.27	**	**	14.0	448.45 ± 156.98	**	514.19 ± 169.78	**	**	23.2
NUpE_cR	**	**	**	1.12 ± 0.04	**	1.19 ± 0.20	**	**	12.3	7.84 ± 0.38	**	7.45 ± 0.74	**	**	−9.6
NUpE_sR	**	**	**	0.88 ± 0.04	**	0.97 ± 0.16	**	**	16.1	5.94 ± 0.45	**	5.79 ± 0.63	**	**	−6.7
NUtE_cR	**	**	**	142.41 ± 37.59	**	118.71 ± 41.83	**	**	1.1	75.52 ±26.39	**	89.74 ± 33.16	**	**	33.4
NUtE_sR	**	**	**	111.53 ± 28.56	**	96.43 ± 34.13	**	**	4.6	57.28 ± 20.20	**	69.59 ± 25.48	**	**	36.3
NTrE	**	**	**	0.78 ± 0.01	**	0.81 ± 0.01	**	**	3.4	0.76 ± 0.02	**	0.78 ± 0.02	**	**	3.1

PH—plant height (cm); SD—stem diameter (mm); LA—leaf area (cm^2^); LDM—leaf dry matter (g); SDM—stem dry matter (g); STDM—shoot dry matter (g); RDM—root dry matter (g); LNC—leaf N content (mg of N kg^−1^); SNC—stem N content (mg of N kg^−1^); RNC—root N content (mg of N kg^−1^); STNC—shoot N content (mg of N kg^−1^); PNC—plant N content (mg of N kg^−1^); (NUE—N use efficiency; NUpE_cR—N uptake efficiency with root N content; NUpE_sR—N uptake efficiency without root N content; NUtE_cR—N utilization efficiency with the content of N in the root; NUtE_sR—N utilization efficiency without the content of N in the root; NTrE—N translocation efficiency. The values in the Lines and Hybrids columns represent the means ± standard deviations of the respective four and twelve evaluated genotypes. C1—statistical differences between strains; C2—statistical differences between lines and hybrids according to the partition of the effects of lines and hybrids; and C3—statistical differences between the hybrids; H%—relative heterosis. Joint ANOVA: genotype (G), nitrogen availability condition (N), and genotype × N availability condition (G × N). Significance levels: ** *p* ≤ 0.01; and ns = not significant.

**Table 2:** Summary of joint and individual ANOVA, means, and standard deviations of physiological traits associated with measurements of gas exchange, photochemical efficiency of chlorophyll, and leaf pigments of lines and diallel hybrids of popcorn cultivated under contrasting conditions of N availability.

Trait	Joint Analysis			High N Condition						Low N Condition					
	G	N	G × N	Lines	C1	Hybrids	C2	C3	H%	Lines	C1	Hybrids	C2	C3	H%
A	**	**	**	23.65 ± 4.34	**	26.15 ± 3.55	**	**	17.5	19.16 ± 3.54	**	17.95 ± 2.88	**	**	−1.4
gs	**	**	**	0.18 ± 0.05	**	0.23 ± 0.05	**	**	33.9	0.16 ± 0.01	**	0.13 ± 0.04	**	**	−4.3
Ci	**	**	**	150.43 ± 22.90	**	158.20 ± 17.77	**	**	2.6	141.46 ± 15.64	**	134.68 ± 24.05	**	**	3.5
E	**	**	**	2.10 ± 0.39	**	2.52 ± 0.56	**	**	28.4	2.11 ± 0.18	**	1.91 ± 0.47	**	**	−9.7
Ci/Ca	**	**	**	0.36 ± 0.09	**	0.43 ± 0.05	**	**	21.9	0.34 ± 0.10	**	0.37 ± 0.09	**	**	20.3
F_v_/F_m_	**	**	**	0.78 ± 0.01	**	0.79 ± 0.08	^ns^	**	1.1	0.79 ± 0.02	*	0.85 ± 0.15	**	**	10.1
Chl	**	**	**	30.65 ± 1.84	**	30.06 ± 4.54	^ns^	**	2.6	24.90 ± 2.47	*	22.37 ± 2.28	**	**	−10.5
Flav	**	**	**	0.70 ± 0.07	^ns^	0.71 ± 0.13	^ns^	**	9.9	0.74 ± 0.07	**	0.78 ± 0.08	**	**	10.2
Anth	^ns^	**	^ns^	0.17 ± 0.01	**	0.16 ± 0.02	**	**	−2.7	0.21 ±0.01	*	0.23 ± 0.17	^ns^	^ns^	26.5
NBI	**	**	**	44.50 ± 5.68	*	43.07 ± 6.94	^ns^	**	−4.1	33.99 ±3.55	**	28.67 ± 3.00	**	**	−18.4

A—net CO_2_ assimilation rate; gs—stomatal conductance; Ci—intercellular concentration of CO_2_; E—transpiration rate; Ci/Ca—ratio between the intercellular and external concentration of CO_2_; F_v_/F_m_—photochemical efficiency of photosystem II; Chl—relative chlorophyll content; Flav—relative content of flavonoids; Anth—relative anthocyanin content; NBI—nitrogen balance index. The values in the Lines and Hybrids columns represent the means ± standard deviations of the respective four and twelve evaluated genotypes. C1—statistical differences between lines; C2—statistical differences between lines and hybrids according to the partition of the effects of lines and hybrids; and C3—statistical differences between the hybrids; H%—relative heterosis. Joint ANOVA: genotype (G), nitrogen availability condition (N), and genotype × N availability condition (G × N). Significance levels: * *p* ≤ 0.05; ** *p* ≤ 0.01; and ns = not significant.

## Data Availability

The data presented in this study are available on request from the corresponding author.

## Supplementary Materials

The following supporting information can be downloaded at: https://www.mdpi.com/article/10.3390/plants12112135/s1, Table S1: Principal component analysis (PCA) for 28 morpho-agronomic and physiological traits evaluated in popcorn genotypes under contrasting nitrogen (N) availability. Table S2: R2 statistics to test the proportional contribution of each source of variation in 28 morpho-agronomic and physiological traits evaluated in popcorn genotypes under contrasting nitrogen (N) availability. Table S3: Analysis of variance and quadratic components for plant growth and physiological traits evaluated in 16 popcorn genotypes under contrasting nitrogen conditions, according to the model proposed by Griffing (1956) for a diallel involving four lines, their F1s, and reciprocal hybrids. Table S4: Chemical and particle-size analysis of the substrate used to evaluate four lines and 12 diallel popcorn hybrids under contrasting nitrogen conditions.; Figure S1: Relative contribution of 28 morpho-agronomic and physiological traits evaluated in popcorn genotypes under contrasting nitrogen (N) availability to the first 2 Principal Components.
