# Supplementary material for: Exploring the Potential of Heterosis to Improve Nitrogen Use Efficiency in Popcorn Plants
Source: Plants (Basel). 2023 May 28;12(11):2135. doi: 10.3390/plants12112135 (PMC10255372; doi:10.3390/plants12112135)
Supplement: Supplementary file 1 [file plants-12-02135-s001.zip › plants-2394846-supplementary.pdf]

Supplementary Table S1. Principal component analysis (PCA) for 28 morpho-agronomic and physiological traits evaluated in popcorn genotypes under contrasting nitrogen (N) availability.

Principal component	Eigenvalue	Variance (%)	Cumulative variance (%)
**High N**			
1	14.78	52.80	52.80
2	9.04	32.30	85.10
3	4.17	14.90	100.00
**Low N**			
1	12.50	44.64	44.64
2	10.10	36.06	80.70
3	5.40	19.30	100.00

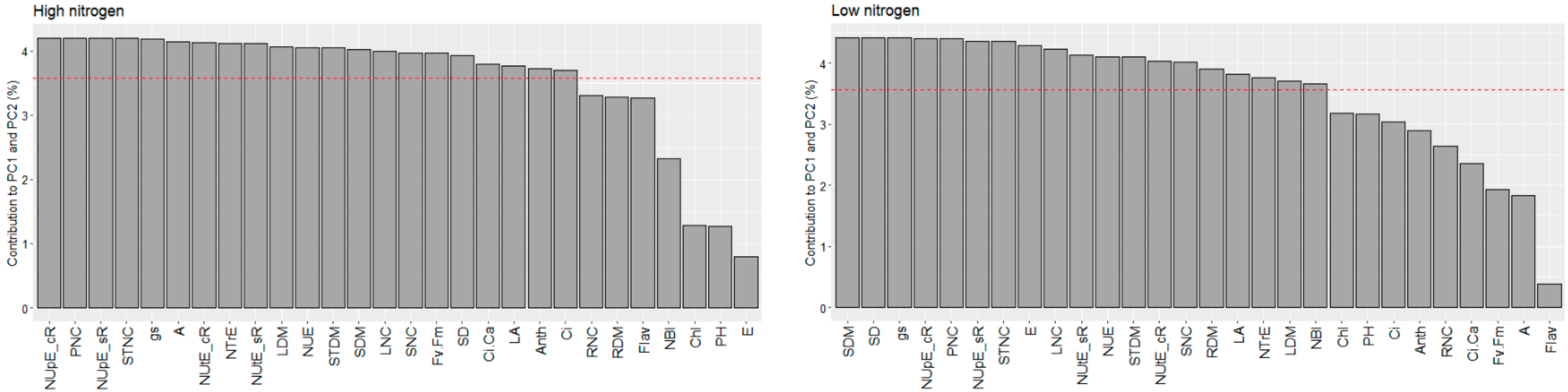

Supplementary Figure S1. Relative contribution of 28 morpho-agronomic and physiological traits evaluated in popcorn genotypes under contrasting nitrogen (N) availability to the first 2 Principal Components. The red line indicates the average contribution. PH – plant height (cm); SD – stem diameter (mm); LA – leaf area (cm2); LDM – leaf dry matter (g); SDM – stem dry matter (g); STDM – shoot dry matter (g); RDM – root dry matter (g); LNC – leaf N content (mg of N kg-1); SNC – stem N content (mg of N kg-1); RNC – root N content (mg of N kg-1); STNC – shoot N content (mg of N kg-1); PNC – plant N content (mg of N kg-1); (NUE – N use efficiency; NUpE_cR – N uptake efficiency with root N content; NUpE_sR – N uptake efficiency without root N content; NUtE_cR – N utilization efficiency with the content of N in the root; NUtE_sR – N utilization efficiency without the content of N in the root; NTrE – N translocation efficiency; A – net CO2 assimilation rate; gs – stomatal conductance; Ci – intercellular concentration of CO2; E – transpiration rate; Ci/Ca – ratio between the intercellular and external concentration of CO2; Fv/Fm – photochemical efficiency of photosystem II; Chl – relative chlorophyll content; Flav – relative content of flavonoids; Anth – relative anthocyanin content; NBI – nitrogen balance index.

Supplementary Table S2. R^2 statistics to test the proportional contribution of each source of variation in 28 morpho-agronomic and physiological traits evaluated in popcorn genotypes under contrasting nitrogen (N) availability.

**Trait**	**Sum of Squares**				**R^2 statistics**		
	**G**	**N**	**G × N**	**Total**	R^2_G	R^2_N	$R^2_{G×N}$
**PH**	1351.56	166.35	610.74	2128.64	**0.635**	0.078	0.287
**SD**	112.27	294.39	54.76	461.41	0.243	**0.638**	0.119
**LA**	46743.95	83920.25	28458.37	159122.57	0.294	**0.527**	0.179
**LDM**	51.28	131.37	27.61	210.26	0.244	**0.625**	0.131
**SDM**	33.27	42.75	19.42	95.44	0.349	**0.448**	0.203
**STDM**	158.28	324.06	81.33	563.67	0.281	**0.575**	0.144
**RDM**	5.51	2.84	3.46	11.82	**0.467**	0.241	0.293
**LNC**	582.17	2322.93	432.16	3337.27	0.174	**0.696**	0.129
**SNC**	585.31	2722.78	471.36	3779.45	0.155	**0.720**	0.125
**RNC**	126.22	258.19	136.31	520.72	0.242	**0.496**	0.262
**STNC**	1776.81	10075.55	1512.41	13364.76	0.133	**0.754**	0.113
**PNC**	2530.05	13559.60	2383.28	18472.93	0.137	**0.734**	0.129
**NUE**	825099.01	3563988.70	565939.84	4955027.55	0.167	**0.719**	0.114
**NUpE_cR**	11.84	972.43	11.34	995.61	0.012	**0.977**	0.011
**NUpE_sR**	8.82	570.26	7.92	587.00	0.015	**0.971**	0.014
**NUtE_cR**	91009.80	32707.18	39096.94	162813.93	**0.559**	0.201	0.240
**NUtE_sR**	57696.23	25439.84	22885.31	106021.37	**0.544**	0.240	0.216
**NTrE**	0.018	0.028	0.015	0.060	0.301	**0.458**	0.241
**A**	646.64	1250.61	466.58	2363.83	0.274	**0.529**	0.197
**gs**	0.087	0.149	0.078	0.314	0.277	**0.474**	0.249
**Ci**	21125.85	9564.15	16386.92	47076.93	**0.449**	0.203	0.348
**E**	8.79	4.91	13.31	27.02	0.326	0.182	**0.493**
**Ci/Ca**	0.281	0.065	0.233	0.579	**0.485**	0.112	0.403
**Fv/Fm**	0.784	0.048	0.294	1.126	**0.696**	0.043	0.261
**Chl**	628.85	1273.00	364.44	2266.29	0.277	**0.562**	0.161
**Flav**	0.489	0.107	0.364	0.961	**0.509**	0.111	0.379
**Anth**	0.172	0.091	0.148	0.411	0.418	0.221	0.361
**NBI**	1118.23	4388.86	1208.65	6715.75	0.167	**0.654**	0.180

Values in bold represents the ones with biggest contribution to their respective Sum of Square. R^2_G – Genotypic contribution to the observed variation in genotypes; R^2_N – Nitrogen condition contribution to the observed variation in genotypes; $R^2_{G×N}$ – Genotype by N interaction contribution to the observed variation in genotypes; PH – plant height (cm); SD – stem diameter (mm); LA – leaf area (cm^2); LDM – leaf dry matter (g); SDM – stem dry matter (g); STDM – shoot dry matter (g); RDM – root dry matter (g); LNC – leaf N content (mg of N kg^{-1}); SNC – stem N content (mg of N kg^{-1}); RNC – root N content (mg of N kg^{-1}); STNC – shoot N content (mg of N kg^{-1}); PNC

– plant N content (mg of N kg^{-1}); (NUE – N use efficiency; NUpE_cR – N uptake efficiency with root N content; NUpE_sR – N uptake efficiency without root N content; NUtE_cR – N utilization efficiency with the content of N in the root; NUtE_sR – N utilization efficiency without the content of N in the root; NTrE – N translocation efficiency; A – net CO2 assimilation rate; gs – stomatal conductance; Ci – intercellular concentration of CO_2; E – transpiration rate; Ci/Ca – ratio between the intercellular and external concentration of CO2; F_v/F_m – photochemical efficiency of photosystem II; Chl – relative chlorophyll content; Flav – relative content of flavonoids; Anth – relative anthocyanin content; NBI – nitrogen balance index.

Supplementary Table S3. Analysis of variance and quadratic components for 28 characters evaluated in 16 popcorn genotypes under contrasting nitrogen conditions, according to the model proposed by Griffing (1956) for a diallel involving four lines, their F1s, and reciprocal hybrids.

Trait	High N condition											Low N condition										
	General combining ability			Specific combining ability			Reciprocal effect			Residual Effect		General combining ability			Specific combining ability			Reciprocal effect			Residual Effect	
	MS	φg	%	MS	φs	%	MS	φrc	%	Value	%	MS	φg	%	MS	φs	%	MS	φrc	%	Value	%
PH	**	1.23	3.07	**	29.568	73.88	**	8.655	21.63	0.571	1.43	**	5.652171	15.39	**	27.9612	76.12	**	3.077	8.38	0.0401	0.11
SD	**	0.398	10.94	**	1.340	36.84	**	1.871	51.43	0.029	0.79	**	0.237307	14.31	**	1.2087	72.87	**	0.208	12.51	0.005	0.31
LA	**	62.333	1.92	**	1593.713	49.01	**	1594.055	49.02	1.521	0.05	**	73.668031	8.17	**	368.207	40.86	**	458.840	50.91	0.505	0.06
LDM	**	0.124	5.11	**	1.428	58.82	**	0.868	35.75	0.008	0.32	**	0.05285	12.19	**	0.25636	59.12	**	0.124	28.58	0.001	0.12
SDM	**	0.051	2.96	**	1.080	63.14	**	0.578	33.77	0.002	0.12	**	0.047863	15.74	**	0.21709	71.39	**	0.039	12.75	0.0004	0.13
STDM	**	0.296	3.85	**	4.813	62.57	**	2.578	33.51	0.006	0.07	**	0.195236	14.82	**	0.87192	66.17	**	0.250	18.95	0.001	0.07
RDM	**	0.017	8.34	**	0.095	46.22	**	0.093	45.33	0.000	0.12	**	0.006572	6.25	**	0.07285	69.34	**	0.024	22.40	0.002	2.01
LNC	**	0.606	3.02	**	7.141	35.52	**	12.204	60.71	0.150	0.75	**	1.063573	6.79	**	11.1186	70.97	**	3.409	21.76	0.076	0.49
SNC	**	0.610	2.06	**	7.920	26.74	**	21.049	71.06	0.041	0.14	**	0.14854	2.72	**	4.40964	80.79	**	0.391	7.17	0.509	9.32
RNC	**	0.053	0.79	**	1.417	21.17	**	5.144	76.83	0.081	1.21	**	0.066015	3.43	**	1.33766	69.50	**	0.484	25.15	0.037	1.92
STNC	**	1.974	2.23	**	23.528	26.60	**	62.721	70.92	0.213	0.24	**	1.109277	16.42	**	1.10928	16.42	**	4.057	60.07	0.479	7.08
PNC	**	2.688	2.09	**	24.826	19.29	**	100.884	78.40	0.286	0.22	**	1.065882	4.16	**	17.4982	68.26	**	6.618	25.81	0.454	1.77
NUE	**	98.204	3.85	**	1597.262	62.58	**	854.874	33.50	1.857	0.07	**	6671.83936	14.82	**	29796.2	66.17	**	8534.953	18.95	30.079	0.07
NUPE_cR	**	0.001	2.09	**	0.008	19.29	**	0.033	78.40	0.000	0.22	**	0.036425	4.16	**	0.59797	68.26	**	0.226	25.81	0.016	1.77
NUpE_sR	**	0.001	2.23	**	0.008	26.60	**	0.021	70.92	0.000	0.24	**	0.037908	6.03	**	0.43536	69.30	**	0.139	22.07	0.016	2.60
NUtE_cR	**	105.270	3.15	**	2320.839	69.52	**	908.248	27.21	4.047	0.12	**	212.348923	13.36	**	926.23	58.28	**	441.565	27.78	9.129	0.57
NUtE_sR	**	68.231	3.32	**	1364.550	66.33	**	622.231	30.25	2.267	0.11	**	132.646943	14.06	**	555.281	58.87	**	252.243	26.74	3.090	0.33
NTrE	**	0.000	0.15	**	0.001	77.79	**	0.000	19.64	0.000	2.42	**	0.000069	9.56	**	0.00051	71.19	**	0.000	13.71	0.000	5.54
A	**	3.498	15.23	**	14.252	62.05	**	4.691	20.42	0.528	2.30	**	0.877728	6.09	**	6.31668	43.81	**	6.799	47.15	0.426	2.95
gs	**	0.001	12.74	**	0.002	62.30	**	0.001	17.81	0.000	7.14	**	0.000012	0.62	**	0.00072	36.89	**	0.001	54.95	0.000	7.54
Ci	**	31.309	5.51	**	317.204	55.82	**	149.046	26.23	70.707	12.44	**	37.472719	4.29	**	530.076	60.65	**	299.354	34.25	7.109	0.81
E	**	0.046	9.76	**	0.252	53.20	**	0.161	33.97	0.015	3.08	**	0.019869	7.03	**	0.14199	50.25	**	0.115	40.61	0.006	2.11

**Ci/Ca**	**	0.000	5.95	**	0.004	58.46	**	0.001	12.08	0.002	23.51	**	0.001184	9.40	**	0.00736	58.45	**	0.004	28.94	0.0004	3.20
**Fv/Fm**	**	0.000	4.28	**	0.004	49.29	**	0.003	40.57	0.000	5.86	**	0.001564	5.28	**	0.01483	50.11	**	0.013	43.40	0.0004	1.21
**Chl**	**	0.363	1.30	**	15.010	53.96	**	10.727	38.56	1.716	6.17	**	0.301963	2.30	**	10.0824	76.82	**	1.938	14.77	0.802	6.11
**Flav**	**	0.002	11.26	**	0.010	49.42	**	0.006	28.67	0.002	10.66	**	0.000677	5.72	**	0.00872	73.70	**	0.002	19.96	0.0001	0.62
**Anth**	**	0.000	2.36	**	0.000	59.84	**	0.000	37.01	0.000003	0.79	ns	-0.0002	0.00	ns	-0.002	0.00	ns	0.002	7.26	0.020	92.74
**NBI**	**	3.389	4.89	**	38.612	55.66	**	14.388	20.74	12.988	18.72	**	1.609887	6.10	**	16.8048	63.65	**	6.519	24.69	1.468	5.56

MS – Mean Square; φg, φs and φrc – quadratic component associated with general and specific combining abilities and reciprocal effects, respectively; PH – plant height (cm); SD – stem diameter (mm); LA – leaf area (cm2); LDM – leaf dry matter (g); SDM – stem dry matter (g); STDM – shoot dry matter (g); RDM – root dry matter (g); LNC – leaf N content (mg of N kg^{-1}); SNC – stem N content (mg of N kg^{-1}); RNC – root N content (mg of N kg^{-1}); STNC – shoot N content (mg of N kg^{-1}); PNC – plant N content (mg of N kg $^{-1}$); NUE – N use efficiency; NUpE_cR – N uptake efficiency with root N content; NUpE_sR – N uptake efficiency without root N content; NUtE_cR – N utilization efficiency with the N content of the root; NUtE_sR – N utilization efficiency without the N content of the root; NTrE – N translocation efficiency; A – net CO_2 assimilation rate; gs – stomatal conductance; Ci – intercelullar concentration of CO_2; E – transpiration rate; Ci/Ca – ratio between the internal and external concentration of CO2; Fv/Fm – photochemical efficiency of photosystem II; Chl – relative chlorophyll content; Flav – relative flavonoid content; Anth – relative anthocyanin content; NBI – nitrogen balance index. Significance levels: * $p \leq 0.05$; ** $p \leq 0.01$; and ns = not significant.

Supplementary Table S4. Chemical and particle-size analysis of the substrate used to evaluate four lines and 12 diallel popcorn hybrids under contrasting nitrogen conditions.

Sample	pH	S-SO_4	P	K	Ca	Mg	Al	Na	H+Al	C	OM	CEC	SB	BS	m	NaSI	Fe	Cu	Zn	Mn	B	N
	H_2O	mg/dm³				mmol$_c$/dm³				g/dm³		mmol$_c$/dm³		%	%	%			mg/dm³			%
**1**	6.7	2	8	0.6	7.9	1.9	0	0.4	3.3	1.3	2.24	14.1	10.8	77	0	3	217.2	0.12	2.3	33.6	0.33	0.18
**2**	6.7	2	8	0.5	7.3	1.9	0	0.7	4.1	1.3	2.24	15.5	10.4	74	0	5	245.0	0.04	2.6	34.0	0.37	0.17
**3**	6.7	2	8	0.6	7.6	1.9	0	0.5	4	1.3	2.24	15	10.6	75	0	5	230.0	0.10	2.5	33.0	0.38	0.17

Extractors: P, Na, K, Fe, Zn, Mn, Cu – Mehlich-1 Extractor; Ca, Mg, Al – KCl Extractor (1 mol/L); H + Al – Calcium Acetate Extractor (0.5 mol/L and pH 7.0); B – Hot water extractor; S – Monocalcium Phosphate Extractor. **Abbreviations:** SB – Sum of Exchangeable Bases; CEC – Cation Exchange Capacity at pH 7.0; BS – Base Saturation Index; m – Aluminum saturation index; NaSI – Sodium saturation index; and OM – Organic matter (C Org × 1,724, Walkley-Black
